# *RRAD, IL4I1, CDKN1A, and SERPINE1* genes are potentially co-regulated by NF-κB and p53 transcription factors in cells exposed to high doses of ionizing radiation

**DOI:** 10.1186/s12864-018-5211-y

**Published:** 2018-11-12

**Authors:** Katarzyna Szołtysek, Patryk Janus, Gracjana Zając, Tomasz Stokowy, Anna Walaszczyk, Wiesława Widłak, Bartosz Wojtaś, Bartłomiej Gielniewski, Simon Cockell, Neil D. Perkins, Marek Kimmel, Piotr Widlak

**Affiliations:** 1 Maria Skłodowska-Curie Institute – Oncology Center, Gliwice Branch, Gliwice, Poland; 2 Department of Clinical Science, University of Bergen, Bergen, Norway; 3 Nencki Institute of Experimental Biology, Polish Academy of Sciences, Warszawa, Poland; 4 Faculty of Medical Sciences, Newcastle University, Newcastle, UK; 5 Institute for Cell and Molecular Biosciences, Newcastle University, Newcastle, UK; 6 Rice University, Houston, USA

**Keywords:** Co-regulation, Integrative genomics, Radiation response, Transcription network

## Abstract

**Background:**

The cellular response to ionizing radiation involves activation of p53-dependent pathways and activation of the atypical NF-κB pathway. The crosstalk between these two transcriptional networks include (co)regulation of common gene targets. Here we looked for novel genes potentially (co)regulated by p53 and NF-κB using integrative genomics screening in human osteosarcoma U2-OS cells irradiated with a high dose (4 and 10 Gy). Radiation-induced expression in cells with silenced *TP53* or *RELA* (coding the p65 NF-κB subunit) genes was analyzed by RNA-Seq while radiation-enhanced binding of p53 and RelA in putative regulatory regions was analyzed by ChIP-Seq, then selected candidates were validated by qPCR.

**Results:**

We identified a subset of radiation-modulated genes whose expression was affected by silencing of both *TP53* and *RELA*, and a subset of radiation-upregulated genes where radiation stimulated binding of both p53 and RelA. For three genes, namely *IL4I1*, *SERPINE1*, and *CDKN1A*, an antagonistic effect of the *TP53* and *RELA* silencing was consistent with radiation-enhanced binding of both p53 and RelA. This suggested the possibility of a direct antagonistic (co)regulation by both factors: activation by NF-κB and inhibition by p53 of *IL4I1*, and activation by p53 and inhibition by NF-κB of *CDKN1A* and *SERPINE1*. On the other hand, radiation-enhanced binding of both p53 and RelA was observed in a putative regulatory region of the *RRAD* gene whose expression was downregulated both by *TP53* and *RELA* silencing, which suggested a possibility of direct (co)activation by both factors.

**Conclusions:**

Four new candidates for genes directly co-regulated by NF-κB and p53 were revealed.

**Electronic supplementary material:**

The online version of this article (10.1186/s12864-018-5211-y) contains supplementary material, which is available to authorized users.

## Background

The biological effects of exposure to ionizing radiation (IR) result from a response to the initial damage of various cellular components. This response includes recognition of DNA double-strand breaks (DSBs) and other DNA lesions, which activate the DNA damage response (DDR) [1], as well as activation of other cellular pathways as the unfolded protein response (UPR) or autophagy [2]. In general, pathways initiated in response to radiation cause reprogramming of the transcriptional network to restore genetic integrity and to eliminate damaged cellular components. Among essential stress-related factors that regulate expression of numerous genes involved in response to ionizing radiation are the p53 and NF-κB transcription factors.

The p53 protein, termed “the guardian of the genome”, is a transcription factor encoded by the *TP53* gene. Regulation of gene expression in response to cellular stress is the main function of p53. Under normal conditions, p53 is functionally inactive due to its rapid degradation by the ubiquitin ligase MDM2, while under stress conditions MDM2-driven degradation is halted, p53 accumulates and gains full competence in transcriptional activation [3]. Moreover, multiple posttranslational modifications of p53 (such as phosphorylation and acetylation) are involved in its regulation [4]. Although many different stress conditions can induce transcriptionally active p53, it appears that two distinct signaling pathways play the major role in p53 activation. One of these is DDR-related activation dependent on several protein kinases, including ATM, ATR, and CHEK2. Another regulatory mechanism is the growth factor/oncogene-mediated signaling pathway that depends on p14ARF tumor suppressor [5]. DDR-mediated activation of p53 results in cell cycle arrest enabling DNA repair (e.g., via activation of CDK inhibitor p21) or apoptosis, if DNA damage exceeds certain “repairable” threshold (e.g., via activation of BAX). However, p53 responsive elements can be found in regulatory regions of several hundred of genes, including factors involved in feedback control loops (e.g., MDM2) and communication with other signal transduction pathways [6, 7]. The p53 protein plays an important role as a tumor suppressor, mostly but not exclusively through its transcription factor activity, thus inactivation of this protein due to *TP53* gene mutation is one of the most common events in human cancers [8]. Interestingly, besides the well-defined role of p53 in DDR and carcinogenesis, p53-dependent mechanisms are also involved in the innate immunity and inflammation [9]. Different types of stress, including radiation, results in p53-dependent activation of Toll-like receptor (TLR) gene expression [10]. Moreover, p53 (together with NF-κB) is involved in the activation of several pro-inflammatory genes in human macrophages and monocytes [11].

NF-κB is a collective name for the transcription factors that work as hetero- or homo-dimeric complexes formed by the NF-κB/Rel family members. Its primary function is a regulation of immune response and inflammation, yet the κB responsive element can be found in regulatory regions of several hundred genes including those involved in apoptosis, activation of cell cycle progression, angiogenesis, and metastasis [12, 13]. Hence, upregulation of the NF-κB pathway is frequently observed in cancer cells, which may contribute to their resistance to anticancer treatments [14]. In resting cells, the NF-κB transcription factors are sequestered in the cytoplasm by association with members of the IκB inhibitory protein family. Pro-inflammatory signals or cellular stress can induce activation of the IκB kinase (IKK) complex, which in turn phosphorylates IκB protein causing its ubiquitination and degradation in the proteasome. Depending on the stimulation, cell type, and cellular context, NF-κB can be activated through different mechanisms [15], yet all of them lead to freeing NF-κB from the inactive complex with IκB, their translocation to the nucleus and binding to the promoter or enhancer regions of target genes. The major pathway primarily activated by pro-inflammatory stimulation, known as the “classical” or “canonical” pathway, typically involves activation of the RelA(p65)/NF-κB1(p50) heterodimer that is determined by IKKβ-catalyzed phosphorylation and subsequent proteolysis of IκBα [16]. The “alternative” pathway involves the IKKα-mediated phosphorylation and processing of NF-κB2(p100), leading to induction of p52 containing NF-κB complexes [17, 18]. Moreover, a number of “atypical” pathways have also been described, including stress/damage-inducible mechanisms of NF-κB activation [19–21]. DSBs activate the NF-κB pathway using an ATM-dependent mechanism. There are multiple pathways of ATM-mediated activation of IKK via NEMO/IKKγ, which leads to phosphorylation and proteolytic degradation of IκBα [22–24]. Furthermore, the ATM kinase has been reported to directly phosphorylate RelA(p65) at Ser-547, which modulates expression of NF-κB-dependent genes [25]. Hence, the RelA(p65)/NF-κB1(p50) heterodimer seems to be the major effector of the atypical NF-κB pathway induced by IR [20, 25]. The radiation-activated NF-κB pathway is also an important element of so-called “sterile inflammation”, a cellular response triggered by damage-associated molecular patterns (DAMPs, or “danger signals”) and resembling pathogen-related inflammation [26, 27].

Both p53 and NF-κB participate in the regulation of expression of numerous genes involved in the same important processes (e.g., cell cycle arrest, DNA repair, apoptosis, immune response, and inflammation). Moreover, pathways dependent on these two factors can be activated by many of the same stimuli, including IR, and the final cellular outcome is determined by the crosstalk between them. Multiple mechanisms of interactions between the p53 and NF-κB have been described. These include genes co-regulated by both transcription factors, which could act as nodes of crosstalk between them. Here we performed an integrative genomics study aimed at discovering new genes potentially co-regulated by both transcription factors in cells exposed to ionizing radiation. To confer dependency of radiation-modulated genes on RelA(p65) and p53, radiation-enhanced promoter binding of RelA and p53 were investigated and gene expression was analyzed also in cells with *RELA* and *TP53* downregulated by siRNA.

## Results

### Activation of p53- and NF-κB-dependent pathways in irradiated cells

**Fig. 1:**
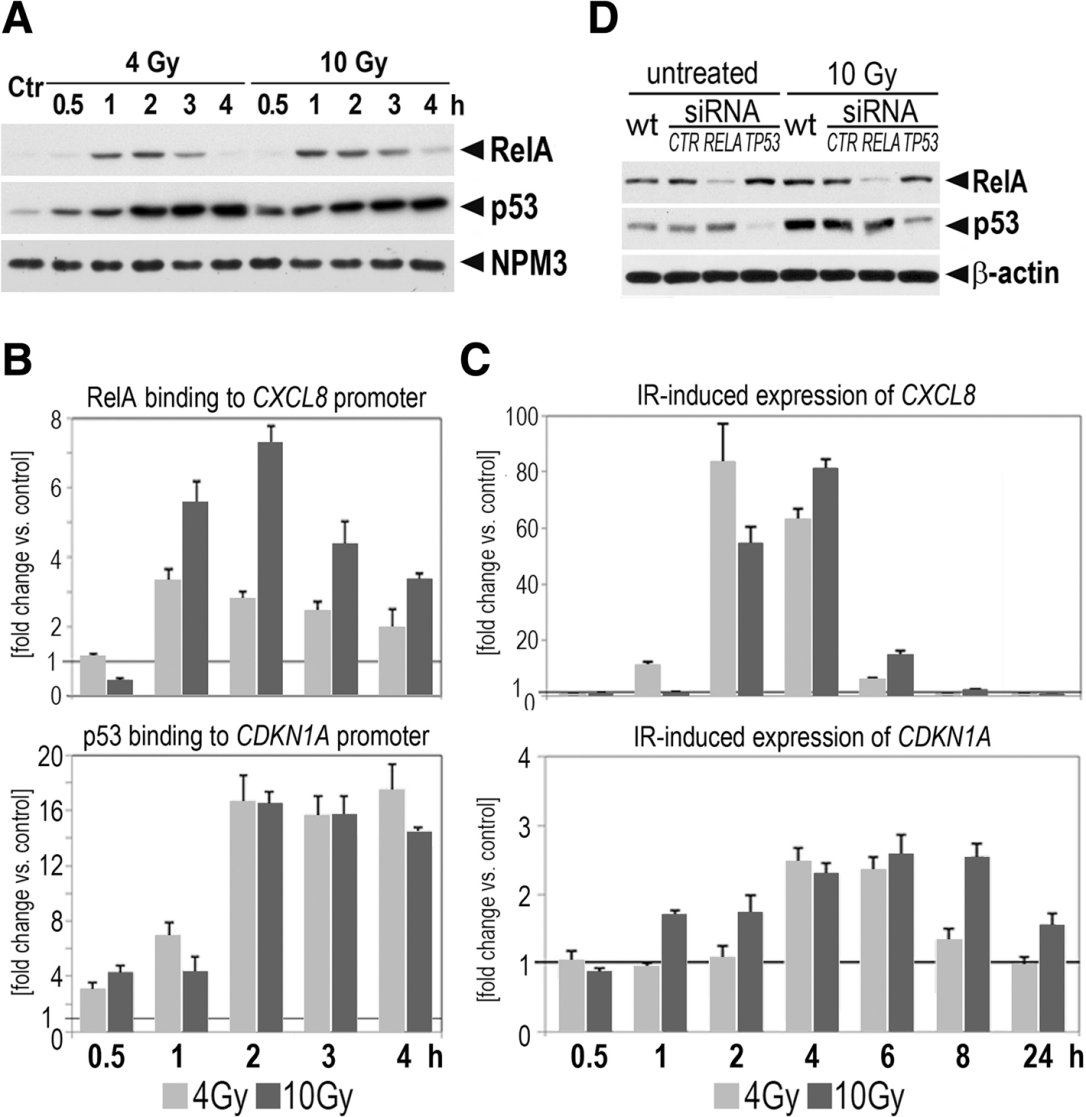
Activation of NF-κB and p53 pathways in U-2 OS cells exposed to a single high dose of ionizing radiation. **a** Kinetics of IR-induced nuclear translocation of RelA and p53 analyzed by Western blot in nuclear extracts (nuclear protein NPM3 was used as a loading reference). **b** Kinetics of RelA and p53 binding in the promoter of *CXCL8* and *CDKN1A* gene, respectively, analyzed by ChIP-qPCR. **c** Kinetics of IR-induced activation of *CXCL8* and *CDKN1A* gene expression analyzed by RT-qPCR. **d** Total levels of RelA and p53 proteins in cells transfected with specific siRNA, either untreated or 4 h. after 10 Gy irradiation, analyzed by Western blot in whole cell lysates

The kinetics of p53 and NF-κB activation were analyzed in cells exposed to 4 and 10 Gy doses of radiation; activation of both pathways was evidenced by nuclear translocation of corresponding transcription factors and their binding to promoters of classical target genes. Increased level of p53 could be observed in nuclear extracts starting 30 min after irradiation (the highest level was noted 2–4 h after exposure), while RelA could be observed in nuclear extracts starting 1 h after irradiation (Fig. 1a). The nuclear translocation of these transcription factors was followed by their binding to respective motifs in promoter regions of target genes: enhanced binding of p53 to the *CDKN1A* promoter and enhanced binding of RelA to the *CXCL8* promoter was seen from 1 to 4 h after irradiation (at either dose) (Fig. 1b). Upregulation of *CDKN1A* expression started 1 and 4 h after irradiation with 10 and 4 Gy doses, respectively, and accumulation of *CDKN1A* transcripts lasted for several hours. In contrast, activation of *CXCL8* was faster and strong accumulation of *CXCL8* transcripts was observed only 2 and 4 h after irradiation (Fig. 1c). It, therefore, appeared that the NF-κB-dependent response was acute in nature, while the p53-dependent response could last for several hours after irradiation. Nevertheless, both types of response appeared to coexist 2–4 h after a single high dose of radiation (4 and 10 Gy), hence these conditions were selected for further experiments. It is noteworthy, that about 50 and < 1% clonogenic survival was observed in cells exposed to 4 Gy and 10 Gy dose, respectively, when long-term effects of irradiation were analyzed (the Additional file 1: Figure S1A). U2-OS cells with downregulated expression of *TP53* or *RELA* were engineered by transient transfection with specific siRNAs; levels of both transcription factors were reduced to 10–30% of their initial levels (both transcripts and proteins) (Fig. 1d and Additional file 1: Figure S2A). It is noteworthy, that the viability of U-2 OS cells was barely affected 6 h after irradiation and it was not markedly modulated by silencing of *TP53* or *RELA* (the Additional file 1: Figure S2B). However, silencing of either gene led to alteration in the cell cycle 24 h after irradiation (the relative number of cells in GO/G1 phase increased in cells with silenced *RELA* and decreased in cells with silenced *TP53*; the Additional file 1: Figure S2C). Moreover, silencing of *TP53* resulted in a decreased number of the sub-G1 fraction (i.e., putative apoptotic cells) 24 h after irradiation (the Additional file 1: Figure S2D). Nevertheless, a good viability of irradiated cells was observed at the time point when the genomics screening was performed.

### Binding of p53 and RelA accompanies radiation-induced upregulation of target genes

**Fig. 2:**
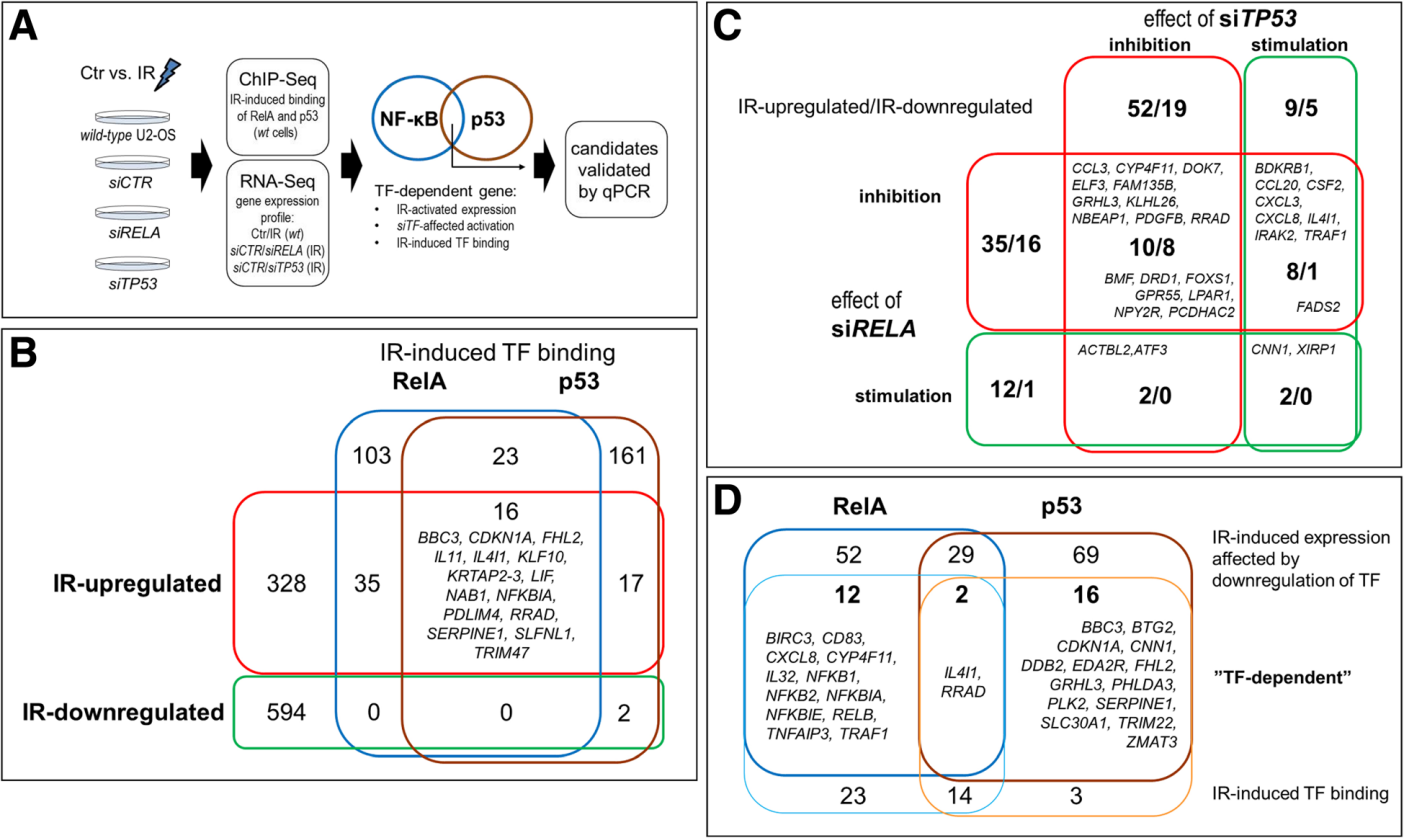
Detection of genes potentially regulated by radiation-activated p53 and NF-κB by integrative genomics. **a** Flowchart of the study. **b** Number of genes upregulated and downregulated by irradiation (at either 4 or 10 Gy dose) in *wt* cells, where IR induced or enhanced binding of either RelA or p53 in regulatory regions (listed are protein-coding genes with the binding of both transcription factors). **c** Number of genes with expression significantly affected by radiation (upregulated/downregulated, respectively; at either dose), whose expression was either inhibited or stimulated in cells with siRNA-silenced *RELA* or *TP53* (listed are protein-coding genes whose IR-modulated expression was affected by silencing of both transcription factors). **d** Number of radiation-upregulated genes with IR-stimulated promoter binding of either RelA or p53 (in *wt* cells), which IR-modulated expression was affected in cells with siRNA-silenced *RELA* or *TP53* (listed are “TF-dependent” genes, i.e., protein-coding genes with expression affected by silencing of transcription factor (TF) where radiation stimulated promoter binding of the corresponding TF)

To search for sets of genes that may be regulated by p53 and NF-κB we first analyzed the global gene expression profile (a flowchart of the study is shown in Fig. 2a). Specifically, gene expression was determined 4 h after a single 4 or 10 Gy radiation dose and then compared to the expression profile of control untreated cells (13,746 analyzed genes are listed in the Additional file 2: Table S2). There were 992 genes with expression affected by IR (at either dose; ca. 40% upregulated). Then, actual radiation-enhanced binding sites of p53 and RelA were identified using ChIP-Seq (2 or 4 h after a single 10 Gy radiation dose). The putative regulatory regions were arbitrarily defined as a region spanning from − 3000 to + 3000 base pairs from the nearest transcription start site (TSS). In general, irradiation of cells resulted in increased binding of p53 in such regions of 219 genes (at either time point), including 33 radiation-upregulated and 2 radiation-downregulated genes. On the other hand, irradiation resulted in increased binding of RelA in putative regulatory regions of 177 genes (at either time point), including 51 radiation upregulated genes (and no downregulated ones). Moreover, there were 39 genes where the radiation-stimulated binding of both p53 and RelA was observed, including 16 genes upregulated by radiation (Fig. 2b). These subsets of upregulated genes consisted of genes potentially regulated by the p53 and/or RelA-containing NF-κB. Additionally, there were several genes where binding of either transcription factor in the potential regulatory region was detected in control not stimulated cells containing 250 genes where binding of both p53 and RelA was detected (data not shown).

### Downregulation of p53 and RelA affects the expression of radiation-induced genes

To address an actual functional importance of p53 and RelA binding, the effect of *TP53* and *RELA* gene silencing on the radiation-modulated expression of target genes was analyzed. The differences between levels of stimulus-modulated expression in cells transfected with control siRNA and *RELA*-specific or *TP53*-specific siRNA were compared for each target gene; differences higher than 50% (i.e., expression ratio > 1.5 or < 0.67) were considered significant. We found that silencing of *TP53* affected the expression of 508 genes, including 116 genes which expression was significantly modulated by irradiation (either dose) in *wild-type* cells; there were 64 radiation-upregulated genes inhibited and 19 radiation-upregulated genes stimulated by *TP53* silencing. On the other hand, silencing of *RELA* affected the expression of 506 genes, including 95 genes where expression was significantly modulated by irradiation (either dose) in *wild-type* cells; among radiation-upregulated genes, there were 53 genes inhibited and 16 genes stimulated by *RELA* silencing. Moreover, there were 31 radiation-modulated genes, which expression was affected by silencing of both *TP53* and *RELA*, including 22 radiation-upregulated and 9 radiation-downregulated (Fig. 2c). These subsets of radiation-modulated genes represent additional subsets of genes putatively regulated by p53 and RelA-containing NF-κB.

### Subsets of p53-dependent and RelA-dependent radiation-induced genes

To reveal final subsets of genes that could be called “transcription factor-dependent” we searched for genes which radiation-modulated expression was accompanied by radiation-enhanced binding of p53 and/or RelA in regulatory regions in *wild-type* cells, and where expression was affected by silencing of *TP53* and/or *RELA* (all previously selected significance thresholds were applied). We found 18 such “p53-dependent” genes; all of them were radiation-upregulated, and all but two (*IL4I1* and *CNN1*) were suppressed in cells with silenced *TP53*. On the other hand, there were 14 genes that could be called “NF-κB-dependent” in irradiated cells; all of them were radiation-upregulated and suppressed in cells with silenced *RELA*. Subsets of p53-dependent and NF-κB-dependent genes are depicted in Fig. 2d and listed in Table 1. GO terms associated with components present in both subsets of genes were identified and compared (see Additional file 2: Table S3). In general, we found similar groups of GO terms associated with both subsets of genes, which included processes related to regulation of gene expression, transport, signal transduction, DNA repair, cell cycle, and cell death. However, there was only one cluster of GO terms - related to regulation of immune functions (cluster BP-1), where the statistically significant difference in the contribution of both subsets was detected (only NF-κB-dependent genes contributed to this cluster). It is noteworthy, that no statistically significant differences in contribution to specific clusters of GO terms were noted if other subsets of radiation-upregulated genes were compared pairwise – genes binding p53 vs. genes binding RelA, and genes affected by *TP53* silencing vs. genes affected by *RELA* silencing (not shown).

**Table 1:** Genes which expression was potentially dependent on RelA-containing NF-κB and/or p53 in cells exposed to ionizing radiation

Gene ID	IR-induced TF binding				Gene expression		Effects of siRNA on IR-induced expression			
	RelA		p53		IR/Ctr fold change (*wt*)		siRELA/siCTR ratio		siTP53/siCTR ratio	
	2 h	4 h	2 h	4 h	4 Gy	10 Gy	4 Gy	10 Gy	4 Gy	10 Gy
*BBC3*	+	+	+	+	2.14*	2.99*	0.86	0.87	0.45	0.42
*BTG2*	–	–	+	–	3.28*	3.92*	0.69	0.68	0.40	0.42
***CDKN1A***	+	+	+	+	2.69*	3.68*	1.16	1.16	0.44	0.46
*CNN1*	–	–	+	–	1.21	1.61*	1.60	1.72	1.49	1.54
*DDB2*	–	–	–	+	1.34*	1.53*	0.94	0.94	0.43	0.48
*EDA2R*	–	–	–	+	2.06*	2.24*	1.10	1.06	0.49	0.49
*FHL2*	+	+	+	–	2.40*	2.92*	1.02	1.06	0.61	0.63
*GRHL3*	–	–	+	–	1.90*	2.23*	1.04	0.87	0.52	0.37
*PHLDA3*	–	–	–	+	1.72*	2.06*	0.91	0.93	0.45	0.40
*PLK2*	–	–	+	+	1.96*	2.22*	1.08	1.09	0.60	0.64
***SERPINE1***	–	+	+	–	2.15*	2.79*	0.98	0.99	0.56	0.56
*SLC30A1*	–	–	+	+	1.81*	1.77*	1.12	1.09	0.73	0.66
*TRIM22*	–	–	+	+	1.56	1.80*	0.87	0.87	0.42	0.44
*ZMAT3*	–	–	–	+	1.81*	1.84*	1.05	1.08	0.48	0.50
*BIRC3*	+	+	–	–	7.04*	17.23*	0.52	0.47	1.41	1.27
*CD83*	–	+	–	–	2.23*	4.03*	0.71	0.62	0.94	0.95
*CXCL8*	+	+	–	–	3.89*	11.53*	0.42	0.37	1.87	1.95
*CYP4F11*	+	–	–	–	1.52*	1.64*	0.63	0.68	0.56	0.74
*IL32*	+	+	–	–	1.35	2.72*	0.48	0.43	1.08	1.06
*NFKB1*	+	+	–	–	2.10*	3.31*	0.72	0.66	1.08	1.13
*NFKB2*	+	+	–	–	1.44	2.21*	0.64	0.63	1.13	1.11
***NFKBIA***	+	+	+	–	1.86*	3.26*	0.65	0.63	1.25	1.28
*NFKBIE*	+	+	–	–	1.65	2.72*	0.59	0.59	1.12	1.08
*RELB*	+	+	–	–	2.60*	4.44*	0.66	0.65	1.06	0.99
*TNFAIP3*	–	+	–	–	3.07*	6.01*	0.70	0.66	1.03	1.02
*TRAF1*	–	+	–	–	2.42*	7.14*	0.47	0.39	2.97	2.06
***IL4I1***	+	+	+	+	1.83*	3.32*	0.48	0.39	1.58	1.23
***RRAD***	+	–	–	+	1.66*	3.56*	0.68	0.65	0.68	0.49

Indicated is RelA and p53 binding in putative gene regulatory regions revealed by the ChIP-Seq in *wt* cells 2 and 4 h after 10 Gy exposure. Gene expression (analyzed by RNA-Seq) is shown as a fold-change 4 h after 4 or 10 Gy dose against untreated *wt* control (asterisks mark *p* < 0.05). The effect of downregulation of either *RELA* or *TP53* gene on a target gene expression is shown as a signal ratio in cells transfected with siRNA specific for *RELA* or *TP53* and control siRNA (siCTR) analyzed 4 h after 4 or 10 Gy dose. Three groups of protein-coding genes are listed: putatively regulated by p53 (upper 14), RelA-containing NF-κB (middle 12), and both transcription factors (bottom 2); genes selected for validation are in bold characters

### Genes potentially co-regulated by p53 and RelA

**Fig. 3:**
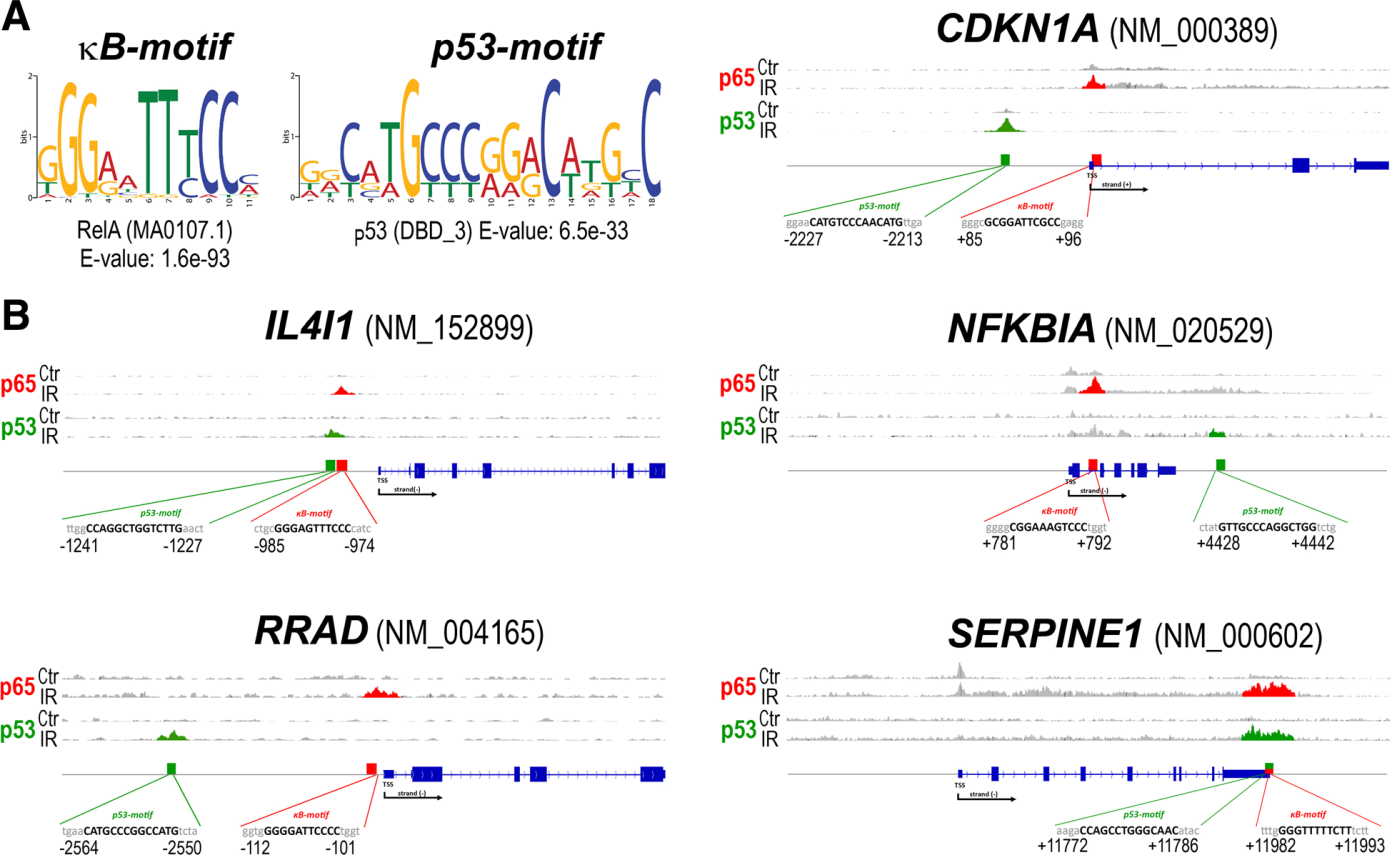
Structure of κB and p53 binding motifs identified in analyzed genes. **a** The most probable common binding motifs revealed after analysis of ChIP-Seq data for all IR-induced peaks (±500 bp from the summits of p65 and p53 binding peaks). **b** Sequence and localization of the most probable motifs in the nearest vicinity of selected genes. Presented are binding regions identified by ChiP-Seq (upper inserts) and localization of analyzed PCR products (red and green bars) together with corresponding gene structures (exons and UTRs are marked with wide and narrow blue boxes, respectively); shown are distances from the Transcription Start Sites of the major transcript variants; red and green marks were used for RelA (p65) and p53, respectively

Genomic profiling allowed us to identify two protein-coding genes, namely *IL4I1* and *RRAD*, where an expression could be called both p53 and NF-κB-dependent. Moreover, the genomics screening revealed a few radiation-upregulated genes that showed radiation-enhanced binding of both p53 and RelA and whose expression was affected by silencing of either *TP53* (including known p53 target *CDKN1A*, and *SERPINE1*) or *RELA* (including “classical” NF-κB target *NFKBIA*) (Table 1). Therefore, five candidate genes: *IL4I1*, *RRAD*, *NFKBIA*, *CDKN1A,* and *SERPINE1* were selected for further validation by quantitative PCR using material from the independent set of experiments. To refine possible binding sites of RelA and p53 in the putative regulatory regions of these genes, potential κB and p53 binding motifs were identified within the binding areas revealed by ChIP-Seq using an informatics approach (the sequence and location of identified motifs are illustrated in Fig. 3). For *IL4I1* and *RRAD* genes both κB and p53 motifs were found upstream of TSSs, while for the *CDKN1A* gene, the p53 motif was upstream of TSS while the κB motif was in the first intron. For the *NFKBIA* gene, the κB motif was found in the first intron but p53 motifs were not identified in binding regions detected within introns by ChIP-Seq; instead, the nearest p53 motif was found downstream of the 3’UTR. Similarly, in the case of the *SERPINE1* gene the obvious κB and p53 motifs were evident in the 3’UTR where a strong binding was also detected.

**Fig. 4:**
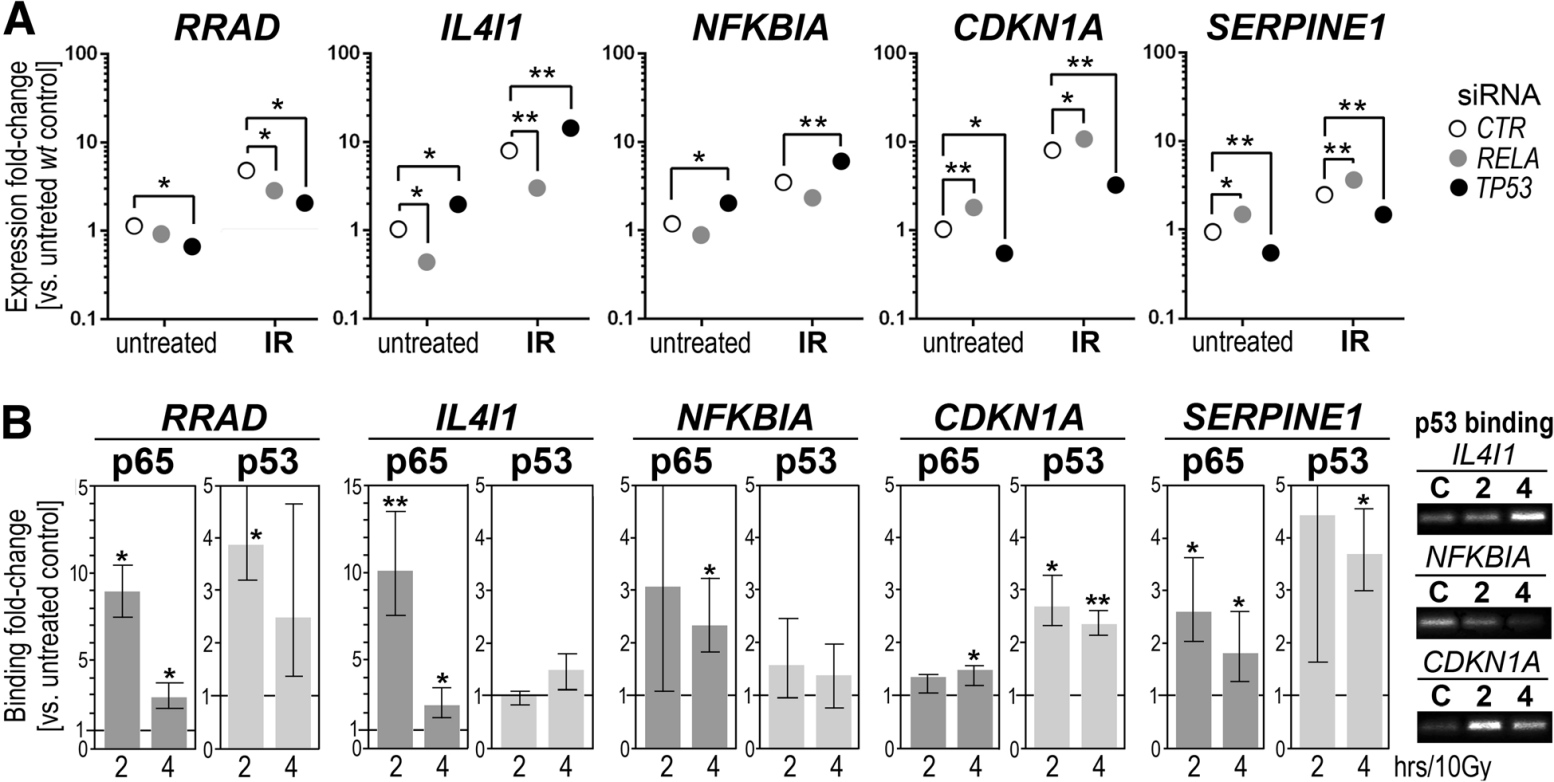
Genes putatively co-regulated by NF-κB and p53; validation of genomics profiling. **a** The influence of silencing of *RELA* and *TP53* on gene expression analyzed by RT-qPCR in the same set of genes 4 h after irradiation with 10 Gy. Expression levels are presented as a fold-change vs. untreated *wild-type* control cells (dots represent mean values); asterisks indicate statistical significance of differences between cells treated with control siRNA and cells treated with siRNA specific for *RELA* or *TP53*: * *p* < 0.05, ** *p* < 0.001. **b** Radiation-stimulated binding of RelA (p65) and p53 in putative regulatory regions of selected genes analyzed by ChIP-qPCR; p53 binding was additionally illustrated by gel electrophoresis of PCR products (C – control, 2 and 4–2 and 4 h after irradiation with 10 Gy). Marked are median, maximum and minimum values of a fold-change vs. untreated control (asterisks indicate statistical significance of differences: *p < 0.05, **p < 0.001)

Further validation experiments confirmed the effect of downregulation of both *TP53* and *RELA* on the expression of all candidate genes (Fig. 4a). The expression of *IL4I1* and *NFKBIA* genes (both “basal” and IR-induced) was inhibited by silencing of *RELA* and enhanced by silencing of *TP53*. The opposite pattern was observed for *CDKN1A* and *SERPINE1* genes, whose expression was inhibited by silencing of *TP53* and enhanced by silencing of *RELA*. Moreover, silencing of either *TP53* or *RELA* inhibited expression of the *RRAD* gene. Furthermore, the actual binding of RelA and p53 to putative regulatory regions was confirmed in irradiated cells in case of *RRAD, IL4I1, CDKN1A,* and *SERPINE1* genes (Fig. 4b). Although only a small induction of p53 binding upstream of *IL4I1* was observed, it was confirmed in several independent experiments (see the right insert in Fig. 4b). On the other hand, IR-induced binding of p53 to *NFKBIA* was not confirmed. To additionally analyze a potential role of RelA in the regulation of *RRAD, CDKN1A,* and *SERPINE1* genes their expression was analyzed in cells stimulated with the TNFα cytokine, where the classical NF-κB pathway is activated (Additional file 1: Figure S3 and Additional file 2: Table S4). We found that cytokine stimulation did not affect the expression of *CDKN1A* and *SERPINE1* genes. However, cytokine treatment resulted in both significant upregulation of *RRAD* and enhanced binding of RelA in its putative regulatory region, which further confirmed a role of this factor in the regulation of *RRAD*.

Results of this validation analysis confirmed a possible direct role of both RelA and p53 in the radiation-mediated regulation of *IL4I1*, *CDKN1A, SERPINE1,* and *RRAD.* However, a direct role of p53 in the regulation of *NFKBIA* was less possible.

## Discussion

Transcription networks depending on p53 and NF-κB, key regulators of cellular response to stress, are among the most studied signaling pathways and multiple mechanisms of communication between these pathways have been described. Interestingly, crosstalk between p53 and NF-κB may be maintained by genes that are common targets of both factors [11, 28]. Such co-regulated genes have the potential to act as nodes of the crosstalk as they could integrate the signaling input from both pathways to produce an integrated output. Hence, knowledge of the genes co-regulated by p53 and NF-κB is essential for understanding the behavior of cells exposed to stress or damage conditions. Sets of genes regulated by either transcription factor have been established in numerous studies, both targeted and genome-wide, and accounts for hundreds or even thousands of components. The meta-analysis of genes actually or putatively regulated by p53 has been recently published by Fischer [7]. According to this analysis about 3660 such genes were reported by different genomics and targeted studies, including a subset of 116 of “the most reliable” genes reported uniformly by at least 6 genome-wide studies. Out of 16 “p53-dependent” genes revealed in the current study, there were 14 genes previously reported by others (including 12 genes from “top 116” subset), while 2 genes, namely *CNN1* and *IL4I1*, were not reported previously (Additional file 2: Table S2). Comparable meta-analysis of genes regulated by NF-κB is not available yet, but a list of about 460 human genes presented by the laboratory of Dr. Thomas Gilmore [https://www.bu.edu/nf-kb/gene-resources/target-genes/] can be used as a useful reference set. Out of 14 “NF-κB-dependent” genes revealed in the current study, there were 10 genes listed in the “Gilmore’s database”; 4 genes (*BIRC3*, *CYP4F11*, *IL4I1*, and *RRAD*) were not reported there (Additional file 2: Table S2). When the overlap of genes regulated by either p53 or NF-κB was analyzed using the abovementioned reference lists, there were 110 genes hypothetically co-regulated by p53 and NF-κB, including 6 genes present in the “top 116” subset (namely *BAX*, *CDKN1A*, *CSF1*, *FAS*, *KITLG*, *PRDM1*). Among the genes whose radiation-upregulated expression was observed in the current study, there were 8 genes listed in the “Gilmore’s reference” of NF-κB-dependent genes, while putative dependence on p53 was reported in at least 3 different studies (namely *CD44*, *CDKN1A*, *CSF1*, *EGFR*, *FAS*, *KITLG*, *NFKBIA,* and *SERPINE1*). However, only 3 of them: *CDKN1A*, *NFKBIA,* and *SERPINE1* were, according to our genomics screen, on the list of putative p53 or RelA targets. Two new candidates revealed in this work - *IL4I1* and *RRAD* were not considered before as co-regulated genes based on available datasets (yet *RRAD* was listed among “top 116” p53-dependent).

Two major modes of co-regulation by two transcription factors are possible – synergistic/additive and antagonistic. The p300/CBP complex is a transcription co-activator essential for expression of genes activated by both p53 and NF-κB, and both transcription factors compete for binding with it. Consequently, NF-κB binding to p300/CBP could suppress the expression of genes dependent on p53, while binding of active p53 to p300/CBP could result in a loss of NF-κB activity [29]. Furthermore, CBP phosphorylated by IKK preferentially binds to NF-κB [30]. Moreover, NF-κB has been reported to enhance expression of MDM2, and consequently negatively regulate p53 [31]. Therefore, an antagonistic mode of action might be the most frequent in the case of genes potentially regulated by both transcription factors, which does not necessarily require a direct role involving binding to regulatory elements in a target gene. Here we noted a set of radiation-upregulated genes whose expression was affected by silencing of both *RELA* and *TP53*, which was one of the hallmarks of putative transcription factor dependency (either direct or indirect). There were genes whose expression was stimulated by both siRELA and siTP53, which suggested negative regulation by both factors, and genes whose expression was inhibited in such conditions, which suggested positive regulation by both factors (the latter group included *RRAD*). Another group of genes was characterized by a putative antagonistic effect of both transcription factors. This included known or putative NF-κB-dependent genes (including *NFKBIA* and *IL4I1,* but also C*XCL8*, *BIRC3, TRAF1*) inhibited by siRELA and stimulated by siTP53, and p53 targets (including *CDKN1A*) additionally stimulated in cells with a reduced level of RelA. An antagonistic effect of p53 and NF-κB is apparently not limited to cells stimulated by ionizing radiation. We have previously reported the enhanced expression of typical NF-κB-dependent genes (including *CXCL8*, *NFKB1*, *REL,* and *TNFAIP3*) in TNFα-stimulated p53-null HCT116 cells [32]. Moreover, we took advantage of the U2-OS experimental model and found 39 genes upregulated after 30 min of stimulation with TNFα cytokine, whose expression was inhibited in cells with downregulated *RELA*. Most importantly, cytokine-induced expression of 29 of such putative NF-κB targets was further stimulated in cells with downregulated *TP53* (the Additional file 2: Table S4), which confirmed the high frequency of the antagonistic mode of interaction. It is most likely that the indirect mechanism of the cross-talk contributed to the apparent negative effect of radiation-activated p53 on radiation-upregulated expression of *NFKBIA* and other NF-κB targets where direct radiation-induced binding of p53 in putative regulatory regions was not observed. However, we revealed radiation-induced binding of p53 and RelA in putative regulatory regions of three genes where apparent antagonistic effects were observed: *IL4I1, CDKN1A* and *SERPINE1*, which suggested a direct role of both transcription factors. It is noteworthy, however, that binding of “inhibiting” factor (p53 for *IL4I1* and NF-κB for *CDKN1A* and *SERPINE1*) was delayed and weaker when compared to “activating” factor. This was in contrast to the RRAD gene, where both factors were “activating” and showed similar strength and time-dependence.

*CDKN1A* is the classical p53-dependent gene coding for protein critical for p53 function – the p21 inhibitor of cyclin-dependent kinases [33]. Thus, potential co-regulation of this gene by radiation-activated p53 and NF-κB could have a major impact on the mechanisms of the cellular response to ionizing radiation. *SERPINE1* gene encodes plasminogen activator inhibitor 1 (PAI-1) involved in hemostasis and proteolytic degradation of the extracellular matrix. A possible role of p53 [7, 34] and NF-κB [35] in the regulation of *SERPINE1* has been already reported. Here we observed that silencing of either *TP53* or *RELA* affected stimulation of *SERPINE1* by radiation and noted radiation-induced binding of p53 and RelA in 3’UTR of this gene. Our study revealed two “novel” candidates for p53 and NF-κB (co)regulated genes: *IL4I1* (antagonistic effect) and *RRAD* (possible synergistic/additive effect). The *IL4I1* gene codes for IL4-Induced Protein 1, an amino acid oxidase active in lysosomal antigen processing and presentation, which is involved in immune-suppressive functions of macrophages and dendritic cells. Expression of *IL4I1* was dependent on the NF-κB pathway activated by pro-inflammatory stimuli [36]. Interestingly, *IL4I1* was among the genes upregulated in hematopoietic cells isolated from mice exposed to total body irradiation. It is noteworthy that radiation-mediated upregulation of *IL4I1* was diminished in cells derived from transgenic p53-null animals, which suggested a role of p53 in the regulation of its expression [37]. The *RRAD* gene encodes a member of the Ras-related GTPase activating proteins family involved in diabetes and glucose metabolism. RRAD protein represses glycolysis through inhibition of GLUT1 translocation to the plasma membrane. It was already reported that *RRAD* is a p53-regulated gene and that under hypoxic conditions p53 represses glycolysis via *RRAD* induction [38]. Furthermore, it has been shown that the direct interaction of RRAD with RelA(p65) and subsequent inhibition of NF-κB nuclear translocation were responsible for the suppression of GLUT1 activation [39]. However, no information on the potential regulation of *RRAD* transcription by NF-κB has been previously reported. Our current data indicated that *RRAD* could be another NF-κB target participating in negative feedback control (similarly to *NFKBIA* and *TNFAIP3*).

In this genomics report, we searched for genes potentially co-regulated by p53 and NF-κB in the general context of cellular response to stress. However, because both transcription factors are involved in many cancer-related processes, including cancer stemness, a potential role of co-regulated genes in these processes is very interesting. Hence, it is important to note that two of them – *RRAD* [40] and *CDKN1A* [41] were reported to affect the activation of stem cell factors OCT4, NANOG, and SOX2. Moreover, the activity of *SERPINE1* was involved in the promotion of the stem cell-like phenotype of cancer cells [42]. Furthermore, *IL4I1* expressed in tumor-associated myeloid cells was involved in immune evasion of cancer cells [43]. Therefore, the identified subset of genes potentially co-regulated by p53 and NF-κB apparently revealed cancer-related properties.

## Conclusions

Four new candidates for genes directly co-regulated by p53 and NF-κB in irradiated cells were revealed, namely *CDKN1A, IL4I1, SERPINE1,* and *RRAD*. *CDKN1A* encodes the p21 protein – the major inhibitor of cyclin-dependent kinases, which has a well-established role in the cellular response to ionizing radiation. Although neither *IL4I1, SERPINE1* nor *RRAD* has an established direct role in the radiation/stress response, they could participate in pathways functionally associated with the systemic response to ionizing radiation. Therefore, potential co-regulation of all four analyzed genes by p53 and NF-κB could have interesting functional implications, which deserve further analysis in a specific gene-oriented study.

## Methods

### Experimental model

Human osteosarcoma U2-OS cells were selected as an experimental model where the activation of a classical p53-dependent pathway [44] and the NF-κB pathway [45] was observed in response to ionizing radiation. The *wild-type* U2-OS cells were purchased from the American Type Culture Collection (ATCC HTB-96) and used as a primary model. Additionally, cells were transiently transfected with 10 nM siRNA (Eurofins) using INTERFERin® Kit (Polyplus Transfection) for 48 h using sequences specific for *TP53* gene (5’-GACUCCAGUGGUAAUCUAC-3′; upper strand), *RELA* gene (5’-GCUGAUGUGCACCGACAAG-3′), or control siRNA (5’-CAGUCGCGUUUGCGACUGG-3′). Cells were grown in McCoy’s 5A medium supplemented with 10% heat-inactivated fetal bovine serum (HyClone) and gentamicin (40 mg/mL; Krka) at 37 °C in a humidified 5% CO_2_ atmosphere.

### Cell treatments

Any treatment started 48 h after inoculation of cells; all steps, with the exception of irradiation, were carried out at 37 °C and 5% CO_2_. For gene expression analysis cells were irradiated with 4 or 10 Gy IR dose at a 1Gy/min dose rate using 6 MeV photons (generated by linear accelerator Clinac 600, Varian), the medium was replaced with a fresh one, and then cells were harvested 4 h after irradiation. For ChIP-Seq analysis, *wild-type* cells were harvested 2 and 4 h after irradiation at 10 Gy. Additionally, cells were incubated with TNFα cytokine (10 ng/ml) (T0157; Sigma) and harvested after 30 min. of stimulation.

### Western blot analysis

Whole-cell lysates were obtained from cells treated with RIPA buffer (1% NP-40, 0.5% sodium deoxycholate, 0.1% SDS in PBS) supplemented with a Complete™ protease inhibitor cocktail (Roche) and phosphatase inhibitor cocktail 2 (Sigma-Aldrich). To isolate nuclei, cells were incubated for 10 min. in an ice-cold lysis buffer (20 mM Tris pH 7.6, 10 mM KCl, 4 mM MgCl_2_, 1 mM DTT, 0.5% NP40, 0.25 M sucrose, and protease inhibitor cocktail), centrifuged at 400 g for 10 min, and resulting pellet (nuclei) was washed twice with PBS by centrifugation. Isolated nuclei were incubated in extraction buffer (20 mM Hepes pH 7.9, 1.5 mM MgCl_2_, 0.2 mM EDTA, 0.4 M NaCl, 25% glycerol, and protease inhibitor cocktail) for 30 min. at 4 °C with gentle shaking. Equal amounts of proteins (25 μg; estimated using the Bradford assay, Biorad) were separated using 10% SDS-PAGE and transferred onto a nitrocellulose membrane (Millipore). The membranes were blocked with 5% nonfat milk in Tris-buffered saline and then incubated overnight at 4 °C with antibodies specific for human p53 (DO-1; Santa Cruz), RelA(p65) (C20; Santa Cruz), actin-β (#4967; Cell Signaling), or NPM3 (ab103779; Abcam). Proteins were visualized after incubation with a peroxidase-conjugated secondary antibody using the enhanced chemo-luminescence kit (Pierce) according to the manufacturer’s instructions.

### Global gene expression profiling

Total RNA was extracted from 1 × 10^6^ cells using RNeasy Mini Kit (Qiagen) and treated on-column with DNase using the RNase-Free DNase Set (Qiagen). Preparation of cDNA libraries and sequencing by Illumina HighSeq 2500 (run type: single read, read length: 1 × 50 bp) were carried out by GATC Biotech AG, Germany (www.bionity.com/en/companies/7128/gatc-biotech-ag.html). Raw RNA-Seq reads were aligned to human genome hg19 using tophat2 [46] with Ensembl genes transcriptome reference. Aligned files were processed using Samtools [47]. Furthermore, reads aligned in the coding regions of the genome were counted using FeatureCounts [48]. Finally, read counts were normalized using DESeq2 [49], then normalized expression values were subject to differential analysis (mean based fold change) and statistical testing using the Student t-test in the R/Bioconductor programming environment. In general, transcripts of 25,369 genes were detected, yet genes with very low signals were filtered out: only 13,746 genes whose signals at any measurement point were above the median value computed for all signals were used for further analyses. Assuming multiple filtering and planned qPCR validation of candidate genes a moderate significance threshold of differences was applied: changes (treatment versus control) were considered significant if signal ratios were > 1.5 or < 0.67 and raw *p*-value < 0.05. The raw RNA-Seq data were deposited in the NCBI GEO database; Acc. No. GSE110762 (part of the SuperSeries GSE110387).

### Gene-specific expression analyses

Total RNA purified as described above was subjected to a two-step RT-PCR reactions. cDNA synthesis was carried out with random hexamer and oligo(dT) primers (1:1) using the RevertAid First Strand cDNA Synthesis Kit (Fermentas); amounts corresponding to 50 ng of RNA were used as templates. The transcript level of selected genes was quantified by RT-qPCR (CFX-96; BioRad) with an application of SYBR Green dye. All reactions were carried out in triplicate at least, and expression levels were normalized according to the *GAPDH* and *HNRNPK* housekeeping genes. The set of delta-Cq replicates (Cq values for each sample normalized against geometric mean of the reference genes) for control and test samples were used for statistical test and estimation of the *p*-values. Sequences of used primers are presented in the Additional file 2: Table S1.

### Chromatin immunoprecipitation

Cells (2 × 10^6^) were inoculated on 10 cm^2^ dish 2 days before experiments. A two-step cross-linking procedure was applied for an efficient fixation of NFκB dimers with DNA [50]. Cells were washed twice with PBS, a solution of DSG (disuccinimidyl glutarate) in 1x PBS was added to the final concentration of 2 mM, and then cells were incubated for 45 min at RT with rotation. Next, cells were washed twice with PBS and the second fixation was performed using 1% formaldehyde for 10 min. at RT. For analysis of p53 binding, cells were fixed only with formaldehyde (as described above). Formaldehyde fixation was quenched by glycine (125 mM final concentration) and then nuclei were isolated using buffers and protocol from iDeal ChIP-seq Kit for Transcription Factors (Diagenode). Chromatin of nuclei re-suspended in 200 μl was sheared using Bioruptor® PLUS combined with the Bioruptor® Water cooler & Single Cycle Valve (at HIGH power setting) with 3 rounds of 5 cycles 30s.ON/30s.OFF; chromatin fragments with approximate length 100–600 bp were obtained. Chromatin immunoprecipitation was carried out using the iDeal ChIP-seq Kit for Transcription Factors (Diagenode) with 3 μl/sample of anti-RelA(p65) polyclonal antibody (C15310256, Diagenode) or anti-p53 polyclonal antibody (C15410083, Diagenode), or without antibody (mock-IP), according to the manufacturer protocol. To verify the efficacy of ChIP reactions promoter regions of human *CXCL8* (for RelA binding) and *CDKN1A* (for p53 binding) genes were amplified by qPCR using specific primers covering the known κB and p53 binding motifs, respectively. Moreover, results of ChIP-Seq were also validated using qPCR (all reactions were carried out in triplicate at least). The set of delta-Cq replicates (Cq values for each ChIP-ed sample normalized against corresponding input value) for control and test sample were used for statistical test and estimation of the *p*-values. Sequences of used primers are presented in the Additional file 2: Table S1.

### Global profiling of chromatin binding sites

Immunoprecipitated DNA fragments and input DNA were sequenced using the HiSeq 1500 system with TruSeq workflow (Illumina). Raw sequencing reads were analyzed according to standards of ChIP-Seq data analysis as described below. Quality control of reads was performed with FastQC software [www.bioinformatics.babraham.ac.uk/projects/fastqc] and low-quality sequences (average phred < 30) were filtered out. Remained reads were aligned to the reference human genome sequence (hg19) using the Bowtie2.0.4 program [51]. Peak detection was carried with the MACS program [52], whereas the outcome was annotated with Homer package [53]. Peak intersections and their genomic coordinates were found using Bedtools software [54]. The input DNA was used as a reference because no sequences were obtained using a mock-IP probe. The significance of differences between control untreated cells and cells subjected to TNFα and IR was estimated using MACS software; the FDR = 0.05 level was selected as the significance threshold. The raw ChIP-Seq data were deposited in the NCBI GEO database; Acc. No. GSE110800 (part of the SuperSeries GSE110387).

### Bioinformatics analyses

Potential κB and p53 binding motifs were identified in selected genes using the MEME Suite package [55]. First, 1000 bp length sequences surrounding summits of all binding regions detected by ChIP-Seq (separately for RelA and p53) were exported and de novo motif discovery was done using the MEME-ChIP tool [56]. Next, specific consensuses established for RelA and p53 were used as patterns in the FIMO tool [57] to scan and locate all κB and p53 binding motifs in all corresponding peaks related with analyzed genes. GO terms associated with analyzed subsets of genes were identified (all three GO domains were used: Biological Processes, Molecular Functions, and Cellular Components). The list of all genes affected by radiation (992 genes) was used as the reference set. To allow direct pairwise comparison of two subsets of genes an original method based on clustering of GO term was used [58]. In order to define groups of functionally related GO terms, each gene was annotated by its corresponding GO terms, taking into account the hierarchical structure of a Gene Ontology graph (only GO terms to which at least two genes were annotated were selected for further analyses). Then, groups of similar GO terms were identified by applying a hierarchical clustering method with G-SESAME similarity measure [59] used to compute the distance matrix T; the number of clusters was arbitrary set to 20. For each cluster, a list of genes that were annotated to GO terms from that cluster was obtained, and contribution of genes present in a cluster was computed as a percentage of all genes detected in a subset. Finally, the significance of differences between subsets regarding gene contribution to clusters of GO terms was estimated using the Fisher exact test.

## Additional files

Additional file 1: **Figure S1.** Clonogenic survival of wild-type U2-OS cells exposed to different doses of ionizing radiation; **Figure S2.** Characterization of U2-OS cells with downregulated RelA and p53; **Figure S3.** The influence of TNFα cytokine on activation of CDKN1A and RRAD genes. (PDF 428 kb)

Additional file 2: **Table S1.** PCR primers; **Table S2.** Complete set of genes analyzed in the manuscript; **Table S3 A** GO terms associated with genes dependent on either p53 or NF-κB, which were used for clustering presented in **Table S3 B**; **Table S3B.** Clusters of GO terms associated with p53-dependent and NF-κB-dependent genes modulated by irradiation; **Table S4** - Gene expression after 30 min incubation with TNFα cytokine; the influence of RELA and TP53 silencing. (XLSX 4530 kb)

## Abbreviations

ChIP: Chromatin immunoprecipitation
DAMP: Damage-associated molecular patterns
DDR: DNA damage response
DSB: Double-strand break
DSG: Disuccinimidyl glutarate
FDR: False discovery rate
IKK: IκB kinase
IR: Ionizing radiation
PBS: Phosphate-buffered saline
RT: Room temperature
GO: Gene ontology
TLR: Tool-like receptor
TNFα: Tumor necrosis factor alfa
UPR: Unfolded protein response
UTR: Untranslated region
