# Supplementary material for: RRAD, IL4I1, CDKN1A, and SERPINE1 genes are potentially co-regulated by NF-κB and p53 transcription factors in cells exposed to high doses of ionizing radiation
Source: BMC Genomics. 2018 Nov 12;19:813. doi: 10.1186/s12864-018-5211-y (PMC6233266; doi:10.1186/s12864-018-5211-y)
Supplement: Supplementary file 1 — Figure S1. Clonogenic survival of wild-type U2-OS cells exposed to different doses of ionizing radiation; Figure S2. Characterization of U2-OS cells with downregulated RelA and p53; Figure S3. The influence of TNFα cytokine on activation of CDKN1A and RRAD genes. (PDF 428 kb) [file 12864_2018_5211_MOESM1_ESM.pdf]

Szoltysek *et al*.: *RRAD, IL4I1, CDKN1A,* and *SERPINE1* genes are potentially co-regulated by NF-κB and p53 transcription factors in cells exposed to high doses of ionizing radiation.

Supplementary File Figures S1-S3

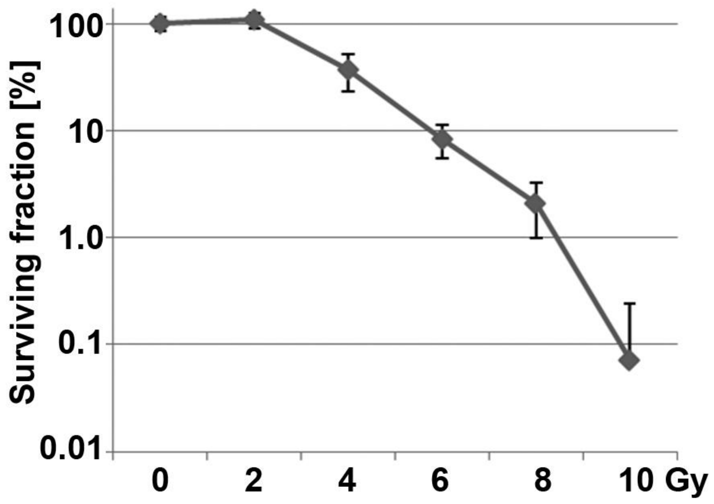

Figure S1. Clonogenic survival of *wild-type* U2-OS cells exposed to different doses of ionizing radiation.

Clonogenic assay. Cells were exposed to a single IR dose (up to 10 Gy), plated on 6-well plates in concentration between 1 x 10^3 up to 10 x 10^4 cells per well (depending on a dose), and then incubated in standard conditions for 2 weeks. After that time medium was removed and cells were washed two times with PBS. Colonies were fixed in 10% formalin solution (Sigma Aldrich) for 30 minutes followed by 0.01% crystal violet staining. Colonies containing more than 50 cells were counted and surviving fractions were calculated.

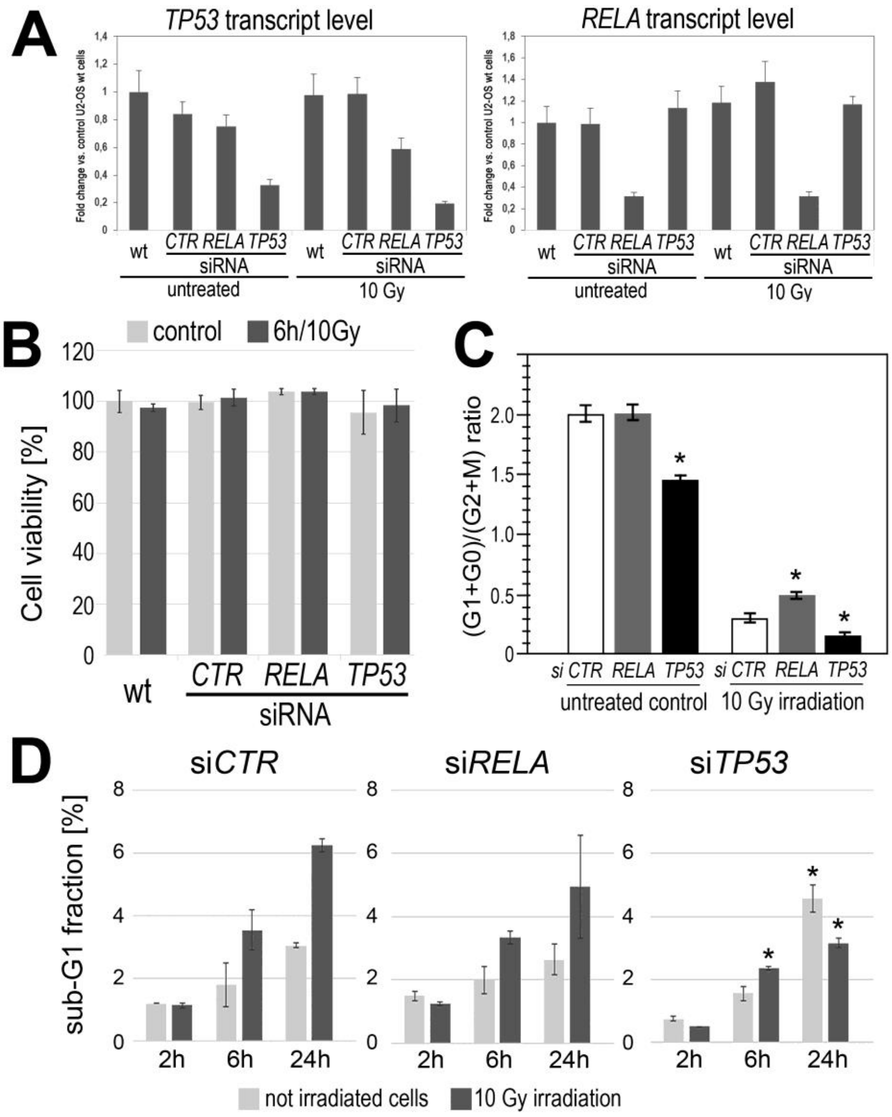

Figure S2. Characterization of U2-OS cells with downregulated RelA and p53. Panel A – Expression of *TP53* and *RELA* genes in cells transfected with specific siRNA, either untreated or 4 hrs. after 10 Gy irradiation, analyzed by RT-qPCR. Panel B – Comparison of the viability of untreated cells, either wt or transiently transfected with specific siRNA, measured by the XTT assay without irradiation (control) or 6 hours after irradiation with 10 Gy. Panel C – The influence of *RELA* and *TP53* silencing on the cell cycle distribution in untreated cells and 24 hours after 10 Gy irradiation; the proportion of cells in different cell cycle phases was measured by flow cytometry and expressed as a (G1+G0)/(G2+M) ratio. Panel D – The influence of *RELA* and *TP53* silencing on the contribution of sub-G1 cell fraction observed 2, 6, and 24 hours after irradiation with 10 Gy and in corresponding not irradiated controls. Marked are the mean values ±S.D. (asterisks denote significance of differences against corresponding siCTR cells, $p<0.05$).

Cell viability assay. Cell viability was analyzed with application of XTT based Cell Proliferation Kit (Biological Industries). Cells were plated on 96-well plates in concentration equal to 1×10^5 cells per well and subjected to the transfection protocol. Then, medium was replaced with the fresh one and cells were incubated with XTT reagent for 4 hrs. Reduction of the tetrazolium salt XTT to orange-color formazan (characteristic for metabolically active cells) was visualized using a microplate reader (BioTek) at a wavelength of 450-500 nm.

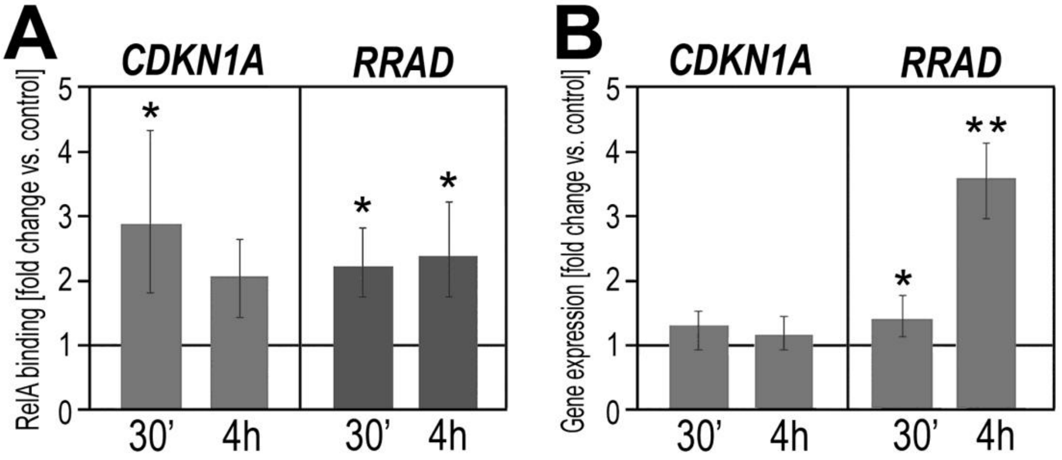

Figure S3. The influence of TNFα cytokine on activation of *CDKN1A* and *RRAD* genes. Panel A – cytokine-induced binding of RelA(p65) in regulatory regions. Panel B – cytokine-induced activation of gene's expression. Analysis by qPCR; asterisks indicate statistical significance of differences between cytokine-stimulated and control cells: * $p<0.05$, ** $p<0.001$.
